# Somatic microsatellite variability as a predictive marker for colorectal cancer and liver cancer progression

**DOI:** 10.18632/oncotarget.3306

**Published:** 2015-01-21

**Authors:** Zalman Vaksman, Harold R. Garner

**Affiliations:** ^1^ Virginia Bioinformatics Institute, Virginia Tech, Blacksburg, VA, USA

**Keywords:** Microsatellites, Colorectal cancer, Liver cancer, Somatic Variability

## Abstract

Microsatellites (MSTs) are short tandem repeated genetic motifs that comprise ~3% of the genome. MST instability (MSI), defined as acquired/lost primary alleles at a small subset of microsatellite loci (e.g. Bethesda markers), is a clinically relevant marker for colorectal cancer. However, these markers are not applicable to other types of cancers, specifically, for liver cancer which has a high mortality rate. Here we show that somatic MST variability (SMV), defined as the presence of additional, non-primary (aka minor) alleles at MST loci, is a complementary measure of MSI, and a genetic marker for colorectal and liver cancer. Re-analysis of Illumina sequenced exomes from The Cancer Genome Atlas indicates that SMV may distinguish a subpopulation of African American patients with colorectal cancer, which represents ~33% of the population in this study. Further, for liver cancer, a higher rate of SMV may be indicative of an earlier age of onset. The work presented here suggests that classical MSI should be expanded to include SMV, going beyond alterations of the primary alleles at a small number of microsatellite loci. This measure of SMV may represent a potential new diagnostic for a variety of cancers and may provide new information for colorectal cancer patients.

## INTRODUCTION

Cancer is a complex disease, and the variety and specificity of treatment options reflect this, differing based on tumor organ origin, cancer stage, malignancy status, previous response to treatment, recurrence and many other factors. To add to this complexity, tumors that originate in the same organ or tissue can respond differently to the same treatment procedure. These challenges have led to a ‘personal’ approach to cancer treatment that relies on a combination of physiology and genomics to determine treatment options [1, 2]. To date the patient specific approach is still very limited because the majority of the known genomic markers are primarily useful for only predisposition screening. One of the few exceptions is a phenomenon called microsatellite instability (MSI). MSI is a pervasive erratic expansion of microsatellites (MSTs), tandem repeats of 1-6 nucleotide motifs, and is associated with approximately 15-20% of colorectal cancers (CRC). MSI is a clinically actionable marker in that treatment options vary in patients with tumors identified as MST unstable (MSI-low or MSI-high) compared to MST stable (MSS) tumors [3, 4]. The identification of MSI, and treatment options associated with its diagnosis, is in part responsible for the drastic improvement in CRC treatment success rate to >65%, as measured by 5-year survival according to the CDC and NCI (http://www.cancer.org/acs/groups/content/@research/documents/webcontent/acspc-042151.pdf). MSI has also been shown be predictive of treatment outcomes and tumor recurrence in other cancers including endometrial, ovarian and breast [3-5].

Unlike CRC, similar genomic markers for liver cancer have not been found. Hepatocellular cancer (HCC) is the 4^th^ most common cancer with ~1 million new cases worldwide and has one of the highest mortality rates of any cancer type [6]. Current 1-year survival rates for liver cancers are <50% and the 5-year mortality rate is ~84% [7] (CDC and NCI website, see above). Several genomic studies have attempted to find a genetic risk factor for HCC, however to date none have been as successful [8, 9]. The known risk factors for liver cancer are exposure to toxins, cirrhosis and uncontrolled diabetes.

Somatic variations (SV), polymorphisms that arise in cell populations, often play a critical role in cellular reprogramming and cancer development [10]. SV resulting from DNA damage or inappropriate nucleotide insertion during DNA replication is often increased during stressed or rapidly dividing cell populations, such as tumors. MSTs are mutational “hot-spots”, meaning they experience a significantly greater rate of somatic variability and population polymorphism than adjacent non-repetitive DNA [11-14]. The unique repetitive genomic configuration of MSTs can lead to the development of complex DNA structures susceptible to polymerase slippage and DNA breaks [11, 15-17]. This results in a distinct mutational profile for MSTs with a bias for indels, as opposed to single nucleotide polymorphisms (SNPs) which are frequently observed in non-repetitive DNA [18]. Although MST expansion of tri-nucleotide (GCC and CAG) repeats have been regularly studied due to their connection to numerous neurological diseases, including Fragile X and Huntington's coria, recent work suggests that MSTs may also exhibit a contraction bias to which mono-nucleotide motifs are most susceptible [14, 19-23]. Many MSTs, especially those in promoter and exonic regions, are under increased selective pressure and therefore MST genomic localization is also important [21, 24-26]. These MST variability trends or biases are significantly altered in cells with impaired mismatch repair (MMR). For example, cells with impaired *MUTYH, MLH1* or *MSH2/6* complexes (associated with familial colorectal cancer, Lynch or Muir-Torre syndrome), two of the three essential complexes required for removal and replacement of incorrect nucleotides, show a significant increase in MSI regardless of genomic localization [19, 27]. For these disorders, although the predominant mutated motif is composed of mono-nucleotides, other motifs, including di- and tetra-nucleotide MSTs, also show an increase in somatic mutations [18, 19, 27].

MSI is a measure of the frequency of altered primary alleles relative to a patient's germline within a select set of microsatellite loci. To date, the only clinically approved test for MSI (Promega, Fitchburg WI) is based on five loci, that is Bethesda markers (BAT-25, BAT-26, NR-21, NR-24 and MONO-27). These MSI markers have been tested for a wide variety of cancers other than CRC, gastric and endometrial tumors, but their global applicability appears to be limited [17, 18, 22]. Expansion of analysis of genomic instability and/or microsatellite instability beyond these 5 loci may yield new markers with more general applicability. The introduction of Next-Gen sequencing (NGS) enabled detailed genomic analysis on a global scale. Over the past two years several papers have compared MSI results obtained from the current clinical test with NGS [19, 27-30]. Results from these publications revealed deficiencies in the current clinical assay in identifying MSI in gastric, cervical and even some colorectal tumors. Our group has recently developed a novel tool that identifies all the sequenced alleles for a given MST locus in a Next-Gen sequenced sample, and was subsequently used to quantify somatic microsatellite variation (SMV) in cell lines with known repair deficiencies [18]. In that study we demonstrated that it was possible to establish a baseline SMV profile in DNA repair proficient cell lines for comparison, and that the SMV profile of cell lines with DNA repair impairment changes in a pathway dependent manner. A comparison of DNA repair proficient cell lines and DLD-1 cells, a CRC MSI cell line, demonstrated an ~70% increase in heterozygotic MSTs loci which was attributed to an increase in mutation rate. The gain in heterozygocity was also found in non-repetitive DNA [16].

Although MSI is presumably a genome-wide phenomenon, the classification of MSI is generally restricted to the small subset of loci that make up the Bethesda markers. Recent genomic studies have argued for an increased emphasis on global neoplastic MST changes to broaden of definition of MSI [18, 19, 27, 29, 30]. This work is the first to test somatic variability of MSTs and non-repetitive DNA sequences in colon and Hepatocellular carcinoma (LIHC) using Next-Gen sequencing. In this paper we show that SMV can be used as an additional measure, yielding information that is not obtained using the current Bethesda markers. The results described here imply a race dependent hypo-variability in CRC patients. Further, MST hyper-variability in LIHC patients may be associated with earlier onset.

## RESULTS

MSI instability is found in approximately 10-20% of CRC tumors and can arise either spontaneously or be associated with hereditary MMR dysfunction. This diagnosis is usually welcome since it provides vital information for treating the patient and is associated with a better patient prognosis. However, recent genome-wide studies indicate that the Bethesda markers may have a higher propensity for false negatives [19, 31]. This underestimation may be due to how global MSI manifests itself. One major assumption is that MSI will be present as a genotypic change, however in a previous publication we reported that MSI can also accompany an increase in the number of non-genotypic alleles present within sequencing data from an individual, or somatic microsatellite variation (SMV) [18]. In this paper we utilize our previously published tool to evaluate SMV trends in CRC patient genomes obtained from The Cancer Genome Atlas, and compare to reported MSI results. Further, we also quantified SMV in patients with liver cancer (LIHC), a cancer not known to have classical MSI.

### Genotype changes in CRC and LIHC patients

We obtained exome sequencing data from 182 CRC patients available from The Cancer Genome Atlas that matched the quality control criteria described in the methods section. For the 182 genomes, all but 9 had a matched tumor and colon/GI control (non-cancer) tissue sequenced as well. For the tumor samples, on average we were able to call 128,589 MSTs per samples (SE ±1,332) with an average read depth of 32 (SE ±4.1) reads per locus called. For the control samples, the mean number of loci called was 126,238 (SE ±1,552) MSTs per sample with a read depth of 33 (SE ±3.3). The mean number of non-MST loci called for tumor samples was 129,101 (SE ±1,620) and 128,593 (SE ±1,613) for control tissue. The average coverage depth and the average number of reads that met our criteria was 36 and 29 (SE ±3.8 and 3.2), respectively. In addition, 82 subjects with liver cancer, LIHC, were available from The Cancer Genome Atlas, 76 of which had both tumor and tissue control samples sequenced. The average number of loci called for the LIHC samples was 123,485 and 126,946 (SE ±1,864 and 2,055) with a depth of 36 and 32 (SE ±4.2 and 4.4) for tumor and control samples, respectively. For non-MST loci, we were able to call 111,733 and 114,549 (SE ±1,295 and 1,465) with a depth of 32 and 33 (SE ±2.9 and 3.7) for tumor and control samples, respectively.

Genomic instability is known to lead to somatically variant DNA sequences that can be detected as changes in genotype. A breakdown of haplotype distribution for CRC cancer and controls shows that 93.6% and 94.3% (SE ±0.13 and 0.09) of the MST loci were homozygotic while 6.4% and 5.7% (SE ±0.13 and 0.09) were found to be heterozygotic (Table 1). In LIHC patients, 95.1% and 94.9% (SE ±0.18 and 0.20 respectively) of the MST loci were homozygotic in tumor and control tissues respectively. As a comparison, the homozygosity rate for non-MST loci that were tested using the same method as that for MST loci (see methods) were found to be significantly lower. In non-MST loci, 98.6% were homozygotic in CRC tumors and controls, and 98.7% for both tissue types in LIHC tumors (Table 1). As anticipated, these results show that MSTs have a higher rate of polymorphism than non-repetitive DNA sequences. These data also suggest a greater discordance rate in MSTs than is found at non-MST loci.

To test the if MSTs do indeed have a greater mutation rate than non-repetitive DNA sequences we measured the discordance rates between somatic and control tissues. Discordance was measured by comparing genotypes for each locus in somatic and control tissues for every individual in our sample set (spreadsheet 1). For each locus that had a difference in genotype we determined if the difference was a loss of allele (shift from heterozygous to homozygous or loss of heterozygosity (LOH)), gain of allele (shift from homozygotic to heterozygotic, aka gain of heterozygosity (GOH)) or if the locus had the same haplotype but a difference in genotype (no allele was the same) [18]. As anticipated, on average, MSTs had a >10-fold increase in genotype discordance rates over non-MST loci (Table 2). The average discordance rate for MST loci was 4.7% (0.15% SE) in CRC patients and 3.5% (0.12% SE) for LIHC patients, whereas non-MST loci showed only 0.39% (0.01% and 0.05% SE) discordance for both CRC and LIHC patients, respectively (Table 2). Both CRC and LIHC patients showed a similar distribution of potential discordance outcomes in MST loci (LOH, GOH or change in genotype but not haplotype). Genotype, but not haplotype, changes made up an average of 51.3% and 54.7% (0.4% and 0.9% SE) for CRC and LIHC patients, respectively, while in non-MST loci this was only 15.45 and 16.65 (0.32% and 0.64% SE) of total discordance loci. An actual change in haplotype, as indicated by LOH or GOH, accounted for only 48.7% and 45.3% of discordant MST loci, while accounting for over 83% for non-repetitive DNA sequences. These results confirm that MST loci have a significantly greater mutation rate than non-MST loci, and that MST associated mutations are maintained in cancer subpopulations. Further, these results show that in these two cancer types, the majority of loci will maintain their haplotype alleles and that of those that do have an altered haplotype, they show an equal likelihood for the gain or loss of a haplotype allele. However, this common method of measuring genomic instability lacks the ability to determine if ‘lost’ alleles (for LOH or loci with genotype but not haplotype differences) disappear completely or are present below the threshold number of supportive reads that would normally be expected of a haplotype allele, suggesting that they are present in a subpopulation of the cells whose genomic content was sequenced.

**Table 1:** Mean (and SE) SMV and somatic variability (SV) in colorectal cancer tumor samples is significantly greater then in control tissue

		Tumor MST			Control MST			Tumor Non-MST			Control Non-MST	
		Mean	SE	Mean		SE	Mean		SE	Mean		SE
CRC patients	Homo-zyg	93.64 #,*	0.13	94.29 *		0.09	98.63		0.02	98.63		0.02
	Hetero-zyg	6.36 #,*	0.13	5.71 *		0.09	1.36		0.02	1.37		0.02
	Multi-alleles	14.30 #,*	0.38	12.79 *		0.36	7.49 #		0.37	6.16		0.34
LIHC patients	Homo-zyg	95.14*	0.18	94.95 *		0.20	98.74		0.03	98.68		0.03
	Hetero-zyg	4.86 *	0.18	5.05 *		0.20	1.26		0.03	1.32		0.03
	Multi-alleles	12.45 #,*	0.92	11.28 *		0.78	7.53 #		0.99	5.68		0.70

# p < 0.01 compared to control tissue for MST or non-MST

* p < 0.01 compared to equivalent tissue for non-MST

**Table 2:** Concordance and types of genotypic changes between tumor and control tissue for CRC and LIHC

		Total loci observed		Percent discordance		% genotype		% LOH		% GOH	
		Mean	SE	Mean	SE	Mean	SE	Mean	SE	Mean	SE
CRC	MST loci	113174	1511	4.69 *	0.15	51.25 *	0.47	22.43 *	0.71	26.32 *	0.83
	non-MST loci	129102	1621	0.39	0.01	15.38	0.32	47.86	1.01	36.76	0.89
LIHC	MST loci	113401	1745	3.51 *	0.12	54.57 *	0.90	23.12 *	0.52	22.31 *	0.63
	non-MST loci	130542	1853	0.39	0.05	16.58	0.64	47.73	1.13	35.69	0.99

* p < 0.05 compared to non-MST loci

### SMV in CRC and LIHC cancer patients

The term SMV here is used to describe the prevalence of minor alleles in MST loci for a given patient. To quantify SMV we analyzed the fraction of MST loci with minor alleles, those alleles that do not contribute to haplotype. The mean rate of minor alleles for CRC tumor MST loci is significantly greater than control tissues (14.3% and 12.8%, SE ±0.4 and 0.4 respectively, p< 0.01) (Table 1). Similarly, for LIHC, tumor samples displayed a greater, but not statistically significant, SMV rate compared to non-tumor control tissue with 12.5% and 11.3% (SE ± 0.9% and 0.8%, respectively) of loci having minor alleles. For both cancers and tissue types, the rate of somatic variability in MST loci was significantly greater (p< 0.01) than in non-MST loci. As shown in Table 1, the fraction of non-MST loci with minor alleles is 7.5% and 6.2% for CRC, and 7.5% and 5.7% for LIHC tumor and control tissues, respectively.

**Figure 1:**
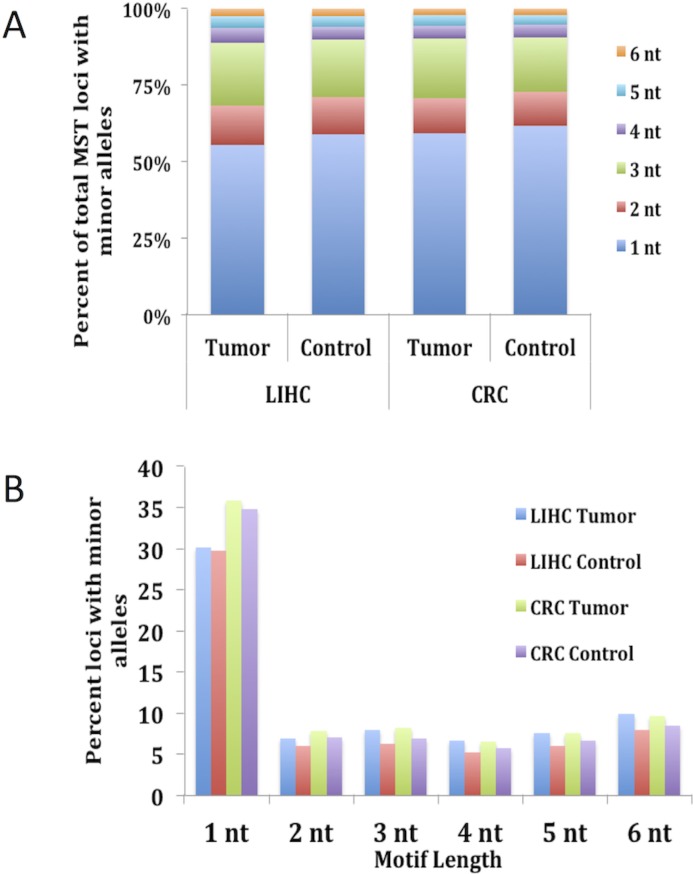
Single nucleotide MSTs show the highest rate of somatic variability and make up over 55% of MST loci with minor alleles The total number of loci with minor alleles in both tumor and control tissue types for each CRC and LIHC patient were calculated and the fractional contribution for each MST motif length was compared. ) shows that single nucleotide motifs make up on average over 55% of the total MST loci with minor alleles while tri-nucleotide MSTs, making up the second highest fraction only make up approximately 21% of the total. Fig. 1B) shows that the reason for this disparity is most likely due to the fact that single nucleotide motifs are 5 – 6 times more likely to have minor alleles than other MST motif lengths. This figure does contain SE bars, however, they are too small to be seen as they are < 2% for 1A and 1% for 1B.

**Figure 2:**
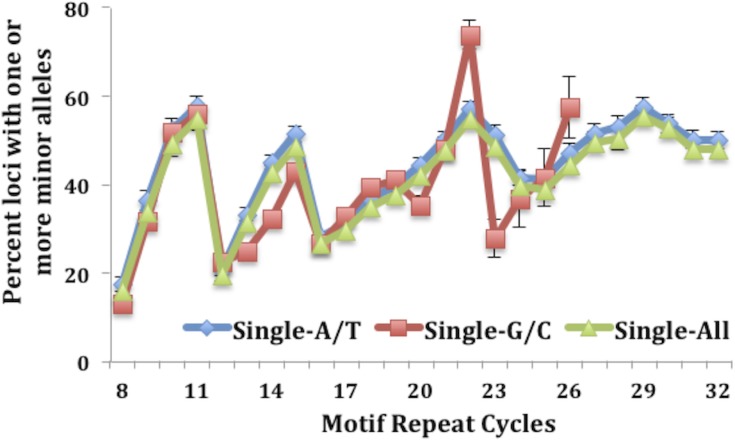
No difference is seen when comparing the fraction of SMV between the two single nucleotide motifs, A/T and C/G runs

Motif length and nucleotide makeup have both previously been shown to play a key role in the stability of MSTs [13, 14, 19]. To evaluate the contribution that the various motifs carry, we determined the MST motif makeup of the loci that have one or more minor alleles. Results depicted in Fig. 1A show that over 55% of MST loci that have at least one minor allele are single nucleotide runs for both CRC and LIHC tumor and control tissues. The next most common MST motif lengths displaying SMV were tri-nucleotide and di-nucleotide motifs, making up ~20% and 12% of the total loci, respectively, (Fig. 1A) for all the cancers and tissue types. These results are significant since single nucleotide repeats make up only 21% of the total MSTs we analyzed while tri-nucleotide repeats make up 36% of the total. Interestingly, using a t-test comparison no significant differences were present for any of the individual motif lengths when comparing the two tissue types within each cancer. To explore the reason for the overabundance of single nucleotide repeats, we calculated the fraction of loci displaying SMV for each MST motif length. The results definitively show that single-nucleotide repeats display a significantly greater rate of SMV (35.8% and 34.8% for CRC and 30.1% and 29.7% for LIHC tumor and control tissue, respectively) than the rates for other MST motif lengths (Fig. 1B). An ANOVA comparison shows no significant difference of the two single nucleotide motifs, A/T and C/G runs, with MST size. (Fig. 2) Taken together these results suggest that single-nucleotide repeats play a disproportionate role in SMV in the two cancer types, and are consistent with previous MST work on major alleles with various cancers, including CRC, by our group and others [19, 23, 32].

**Figure 3:**
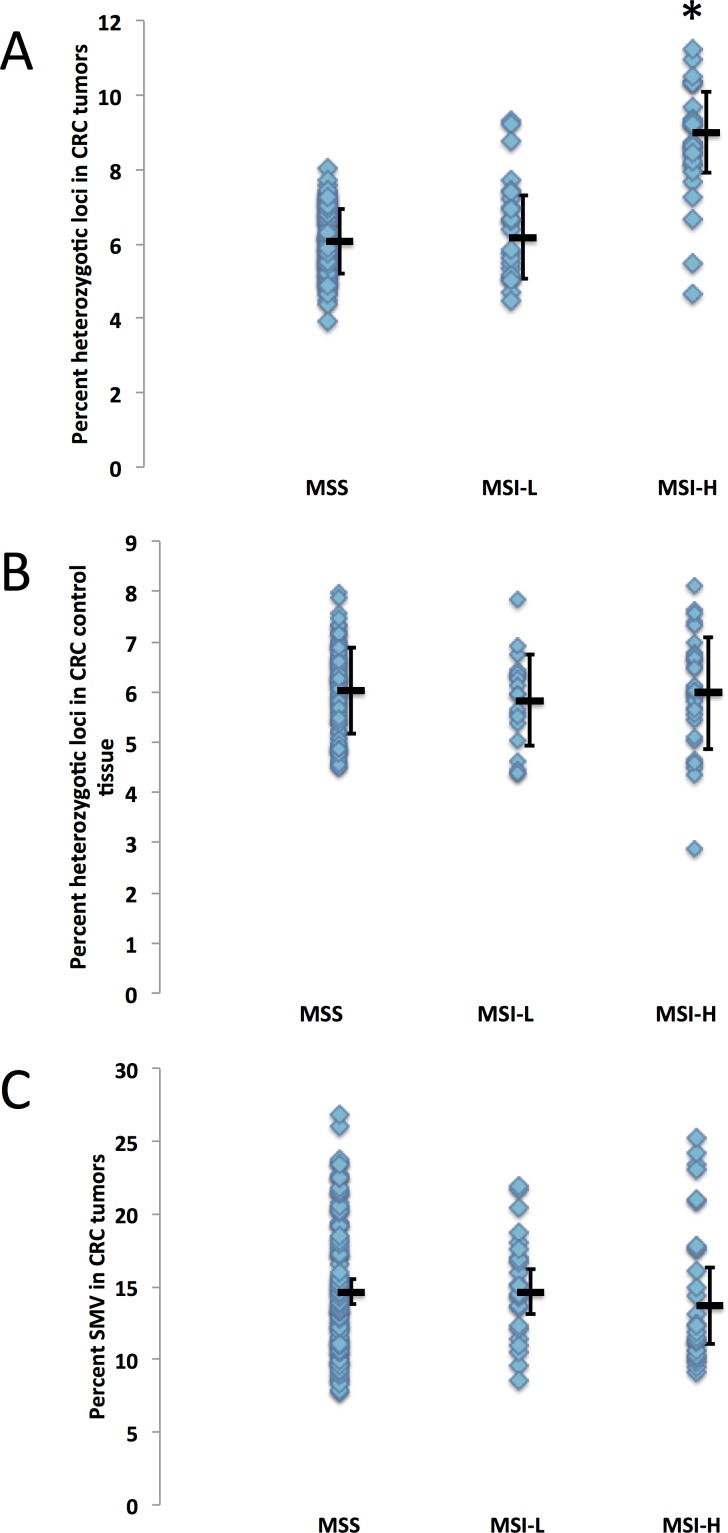
Microsatellite instability is not correlated to SMV in colorectal cancer CRC patients were grouped by MSI status (classified as MSI-stable (MSS), MSI-L or MSI-H) and analyzed for differences in genomic stability by comparing A) fraction of heterozygotic loci in tumor tissue, B) fraction of heterozygotic loci in control tissue and C) SMV in tumor tissue. The MSI-H group displayed a significant increase in heterozygosity as compared to MS-S and MSI-L group (* - p < 0.01, ANOVA followed by a Fishers PLSD test) while no statistical difference was found when observing SMV in tumor tissues (ANOVA p > 0.24).

### SMV in CRC

MSI is most commonly associated with hereditary CRC, therefore MSI testing is commonly conducted on these patients. Within the CRC dataset we analyzed, MSI metadata testing results are given for 155 patients. Of the patients for which the data is supplied, 102 are considered MS-S (MST stable), 24 are MSI-L (with 1 – 3 of the 5 loci showing different primary alleles, and 29 were found to be MSI-H (with 4 or more of the loci showing different primary alleles). Since MSI is considered a genome wide phenomenon, we hypothesized that patients testing MSI-H may also show an increase in SMV, that is in addition to acquiring/loosing primary alleles, they would show an increase in the number of loci that have robust minor alleles. We compared MSI status with haplotype and found that heterozygosity was significantly increased in MSI-H tumors (9.0%) as compared to CRC tumors testing MSI-L and MS-S (6.1% and 6.2% MS-S and MSI-L respectively, Fig. 3A). This was not the case for control tissue, where no significant difference emerged between the three MSI groups (Fig. 3B), confirming that heterozygosity changes were predominantly introduced in MSI-H tumors. We next compared the fraction of loci with minor alleles with MSI status in tumor samples and found no significant difference between the MS-S, MSI-L and MSI-H subgroups (14.6%, 14.7% and 15.1% respectively, Fig. 3C). These results suggest that haplotype, but not SMV measures, can be used to predict tumor MSI status in CRC patients.

**Figure 4:**
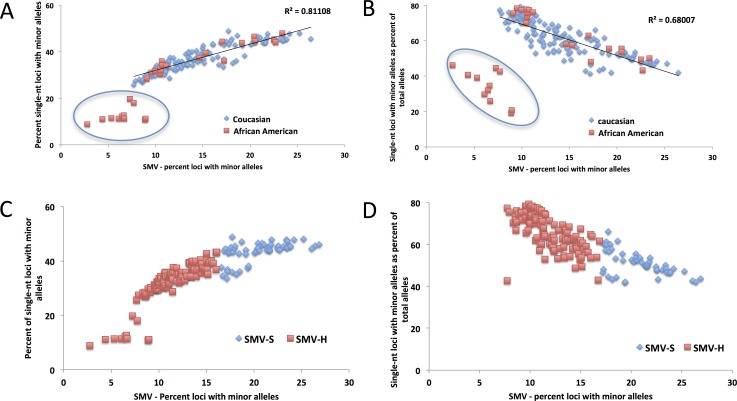
The distribution of the contribution of single nucleotide SMV to overall SMV in CRC patients A) A linear fit of the total SMV as a function of single nucleotide SMV distribution and B) total SMV as a function of single nucleotide as a fraction of total SMV. In both figures a clear set of outliers entirely consisting of 32% of the total African American (orange squares) CRC patient population is encircled. C) and D) show the same distributions with the binomial fit inflection point used as a cutoff between SMV-high (blue diamonds) and SMV-stable (orange square) patient groups.

**Figure 5:**
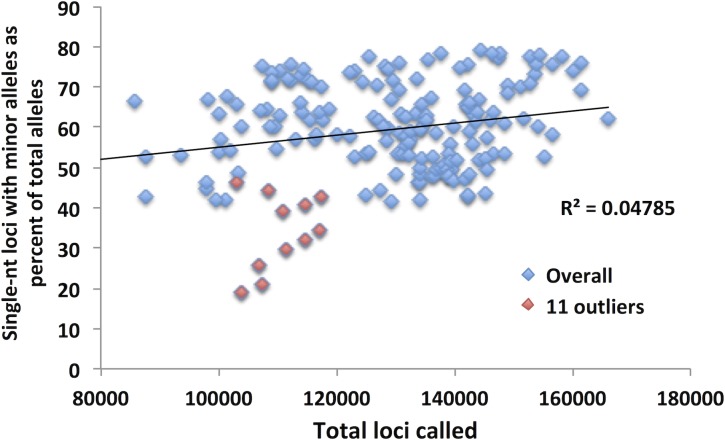
The total number of loci called for the 11 outlier African American CRC patients does not explain their low SMV Although the mean for the 11 patients was lower, the total number of loci called was within the distribution for all the CRC patients.

In the previous section we demonstrated that single-nucleotide repeats contributed disproportionally to overall SMV in both CRC and LIHC patients. Further, a comparison of the fraction of single-nucleotide loci with minor alleles and overall SMV revealed a significant positive correlation (Fig. 4A). To confirm that this disproportionate contribution does not bias overall SMV, we evaluated the relationship between the fraction of single-nucleotide motif loci contribution to the overall SMV rate. Surprisingly, the results yielded a negative correlation of the two factors (Fig. 4B); meaning that as the overall SMV rate increases the influence of single-nucleotide loci on the total SMV is reduced. Two aspects should be noted in both of the described correlations: first is that a binomial is a much better fit than a linear regression, with a biphasic inflection point at 16% SMV rate (Fig. 4C and D, after removal of outliers); second is that the small group of 11 outliers, the subset that were encircled in Fig. 3A and B, consisted solely of African American/Black CRC patients and represents 38% (11 of 38) of African American/Black subjects analyzed in this study. The 11 patient specimens consisted of 5 males and 7 females, and although they were acquired in 5 different centers (Christiana Healthcare, Candler, International Tissue Consortium, University of Pittsburgh and Fondazione-Besta), all were sequenced at Baylor College of Medicine, thus minimizing technical sequence acquisition bias. To eliminate differences in coverage, as the reason for the outliers, we compared the total number of loci called for each sample to the fraction that single-nucleotide motifs contributed to the total SMV and found no significant correlation (Fig. 5). Further, no difference was observed when comparing mean coverage per called MST locus for the 11 patients (23.3 (2.2 SE) for the 11 patients and 24.8 (1.3 SE) for the remaining CRC population tested. These results suggest a potential predisposition for CRC in African American patients that is not currently known or tested in this patient population. Interestingly, MMR may be associated with CRC in this population, however, none of these 11 patients have been tested for known MMR deficiencies (80% of the untested CRC subjects were African Americans).

### SMV and cancer onset – CRC and LIHC

**Figure 6:**
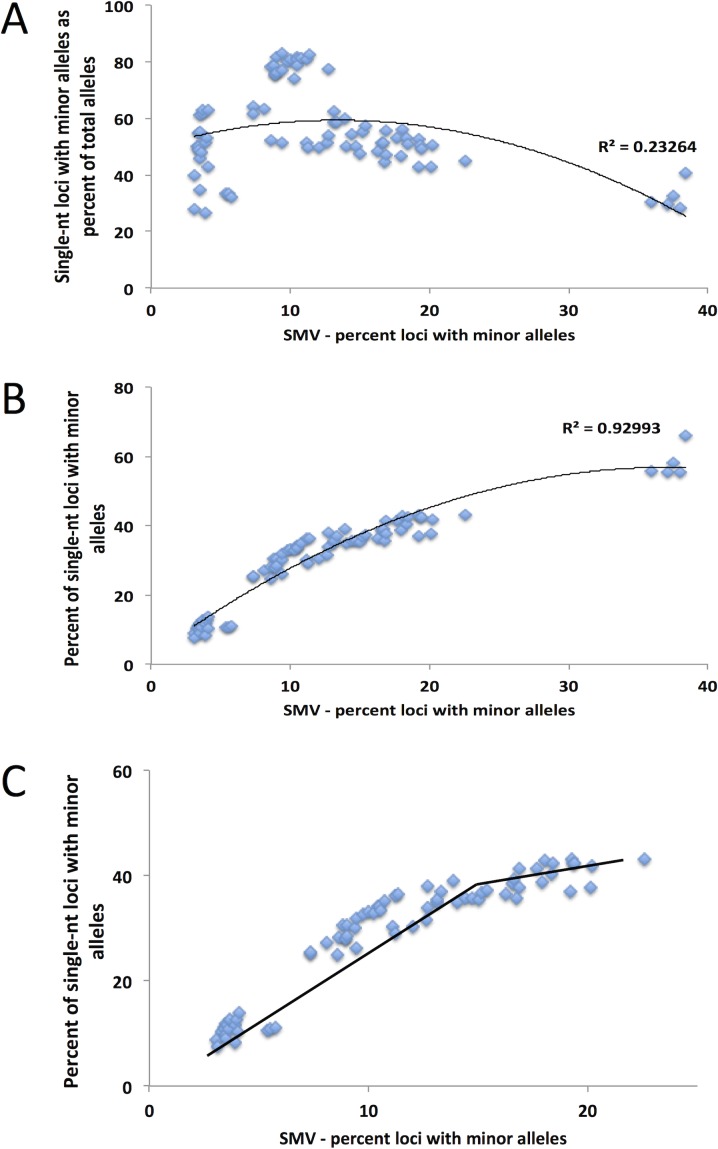
A binomial distribution is the best fit model for the comparison of single nucleotide SMV and total SMV for LIHC patients, with the inflection point serving as a break point between SMV-high and SMV-stable A) A binomial fit for the fractional contribution of single nucleotide motifs to total SMV. B) The fraction of single nucleotide SMV as compared to total SMV. C) When the 5 statistical outliers (z transformation and Grubbs test) are omitted from the distribution, the inflection point at 14% SMV, presented by the break in the line, serves as the break for distinguishing between SMV-high and SMV-stable.

As with CRC patient data, when we analyzed the LIHC samples, single-nucleotide loci made up over 55% of the total loci with minor alleles (while they make up only 21% of the total MSTs called) in both tumor and tissue control samples (Fig. 4A and B). Also, as with CRC patients, we used this data to determine a cutoff for SMV-high and SMV-stable groups. A regression analysis, results of which were plotted in Fig. 6A, shows a reduction in the fraction of single-nucleotide loci that make up the overall number of loci with minor alleles. These results are consistent with CRC patients. As seen in Fig. 6A and also 4B, there are 5 patients that are clearly outliers, however, unlike with the CRC data, the make-up of this group includes 4 Caucasians and one individual of African descent; 3 males, 2 females; and 4 of the 5 had a predisposition (either alcohol abuse or infection). Further, all the samples were sequenced in the same center. Therefore, unlike the CRC individuals, there is no indication of why these samples might be outliers; however, they were removed from the following analysis.

**Figure 7:**
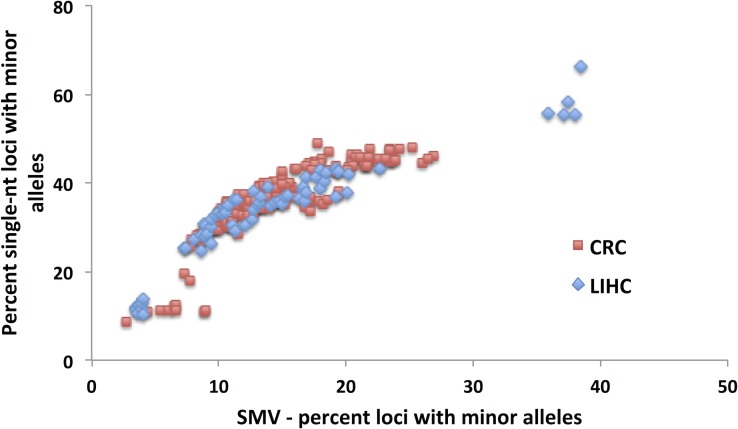
No difference in the distributions of total SMV as a function of single nucleotide SMV between CRC and LIHC patients An overlay of CRC and LIHC patient data is presented.

A comparison of the fraction of single-nucleotide loci with minor alleles to the overall SMV rate, as depicted in Fig. 6B, shows a concurrent increase in the rates of both; a positive correlation, in a biphasic manner, similar to CRC data. When we overlayed and statistically compared the patient data from both cancer types, CRC and LIHC, we found no difference in the distribution (Fig. 7). We found the point of inflection to be at 14%, which we used as the cutoff for classifying an individual as SMV-stable or SMV-high. Using the inflection points for both CRC and LIHC tumors, we compared age of onset for SMV-high and SMV-stable patients. For CRC tumor samples, no significant difference was found in the age of initial diagnosis between SMV-stable and SMV-high (66 and 65 years of age, respectively). Similarly, age of diagnosis was not significantly affected by MSI status (data not shown) in this dataset. However, for LIHC a significant difference did emerge between the two subgroups with the mean age of diagnosis for SMV-stable as 66.0 (±1.5) and 59.1 (±2.9) for SMV-high (Table 3). Similar results were found when the cut-offs were used with non-tumor control tissue sequencing data for both LIHC and CRC patients (Table 3). Again, only SMV-high LIHC patients were found to have a significantly lower age of onset as compared to LIHC SMV-stable. These results indicate that SMV may be a valuable measure of MST instability and may serve to expand the role of MSI to other cancers, in addition to CRC and endometrial cancers.

**Table 3:** SMV-H in both tumor and controls tissue is correlated to lower age of onset for liver cancer, but not for colorectal cancer

	Cancer	SMV-S Mean (SE)	SMV-H Mean (SE)	T-test (p <)
CRC	Tumor tissue	66.0 (1.1)	65.0 (1.7)	0.28
	Control tissue	63.1 (1.9)	65.8 (1.1)	0.14
LIHC	Tumor tissue	66.0 (1.5)	59.1 (2.9)	0.04
	Control tissue	66.7 (1.4)	58.4 (3.0)	0.02

## DISCUSSION

The importance of MST instability in cancer cannot be overstated. Identification of individuals with impaired MMR, or Lynch-syndrome, leading to early onset MST-unstable CRC or endometrial cancer has lead to earlier detection and a significant decrease in mortality in this subset of CRC patients [3, 4]. Although several genomic studies have found gene markers associated with liver cancer tumors, none of these are informative for age of onset or treatment [33-36]. The importance of finding a marker for predisposition or treatment is underscored by the fact that liver cancer has the second highest mortality rate of all cancers; and according to the National Cancer Institute, approximately 30% of patients have no predisposition markers (www.cancer.gov/cancertopics/pdq/treatment/adult-primary-liver). In this paper we examined somatic variation at microsatellites, to assess its utility in predicting early onset of LIHC or CRC.

MSI is currently defined based on markers found that are specific to CRC and using molecular identification methods. With the reduction in cost in genomics and its increased use in clinical settings, an expansion of how MSI is defined may allow it to be used as a predictive tool for more cancers. To use our measure of SMV as a diagnostic tool, a threshold for instability was identified, by which we were able to differentiate SMV-stable and SMV-unstable populations, similar to the utility of the Bethesda markers for distinguishing between MSI-stable and MSI-high CRC. We defined SMV status based on overall SMV rate as a product of single-nucleotide SMV. This method was partially based on previous work by Yoon et al [19] in which they used single nucleotide repeat genotype changes as a cut-off measure to determine MSI status based on Next-Gen sequencing. For our study, the cutoff for determining SMV-high or SMV-stable patient populations was selected at the point of inflection in the binomial distribution (Fig. 4C and D, and 6C) for each type of cancer. This cutoff was selected because it was associated with a stabilization of single-nucleotide runs while overall SMV, as well as the SMV for other motifs, was still increasing. Mutation rates for single-nucleotide MSTs are known to be significantly higher than other motifs, but here the rates plateaued at only 40-50%, lower than anticipated. We speculate that this point may represent a change either in mechanism associated with mutation accumulation or the maximum SMV rate for single nucleotide repeats.

Using the cutoffs to distinguish SMV-high and SMV-stable patients, we assessed age of first diagnosis for each cancer type. Counter to several previous studies on MSI [29, 37] in this population of CRC patients, MSI status was not associated with early initial diagnosis. Using the SMV measure we found the same result; SMV-high was not associated with an earlier age of diagnosis. However, LIHC SMV-high patients in this study were, on average, diagnosed 6 – 7 years earlier than SMV-stable patients. Although onset and diagnosis can be separated by years in LIHC, the fact that cancer stage at diagnosis did not differ significantly between the two SMV groups suggests that the onset data would parallel detection. It would have also been beneficial to compare SMV rates with the outcomes of various treatments, especially for LIHC patients, however the variability in the treatments used and inconsistent outcome reports prevented that comparison.

**Figure 8:**
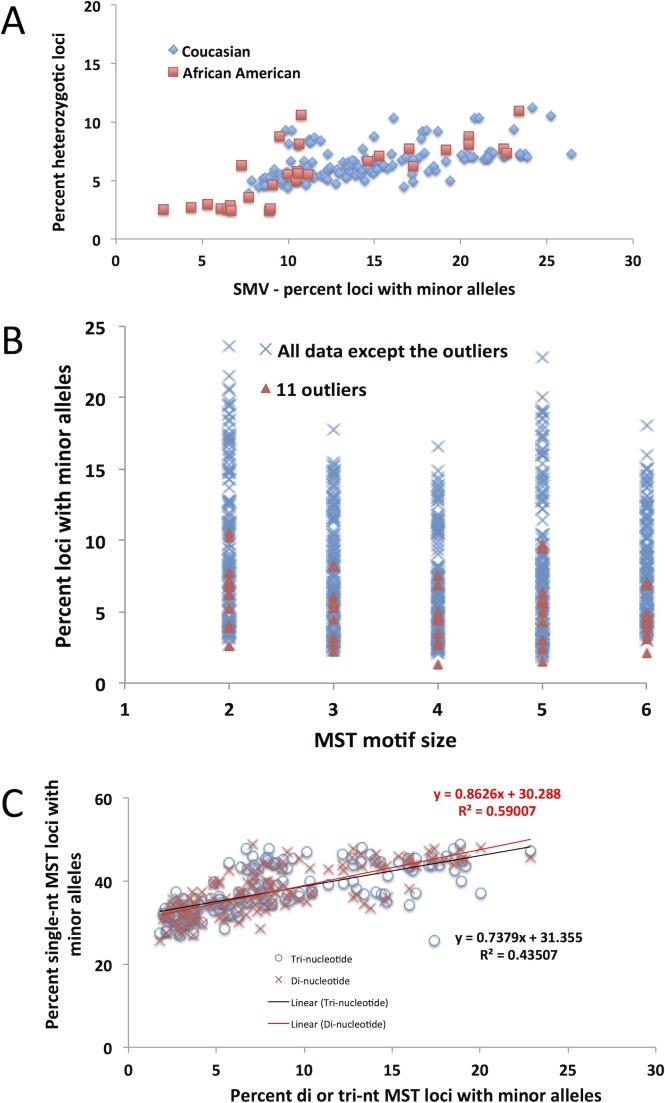
The 11 CRC outliers based on the previously described distribution are not outliers as identified using any other MST motifs A) A distribution of the fraction of heterozygotic loci as a function of total SMV rate. Encircled are the 11 outliers who, as in Fig. 4A, remain outliers as a function of both parameters, not just SMV. B) The 11 outliers (orange triangles) are not outside of the distribution for any other motif length, other than single nucleotide repeats. C) The distribution of single nucleotide repeats as a function of SMV at di- and tri-nucleotide repeats. The slope of the regression line is below one, meaning that SMV at di and tri nucleotide repeats increases at a higher rate than at single nucleotide repeats.

MSI is defined as an increase in MST primary allele variation, however our use of SMV as a measure of MST stability indicates that both increases and decreases in MST variation rates can be informative. In this study hypo-variability was found to be associated with race, as a specific marker for African American CRC patients. A subset of ~30% of African American patients in this study, show a distinct pattern of MST stability with low SMV and lower heterozygosity rate. These 11 are in contrast to MSI-high patients which have a significantly higher heterozygosity rate compared to the rest of the CRC population (Fig. 8A and 3A). Rather, the hypo-variability found in these 11 CRC patients is mainly due to the low single nucleotide SMV rate, as these patients would not be identified as outliers if we used SMV rates for other motif lengths (Fig. 8B) even though they show a very low SMV rate at all other MST motif lengths.

One of the most unexpected observations is the inverse relationship between the contributions of single nucleotide SMV to the total SMV when regressed against total SMV (Fig. 4B and 6A). This means that as the overall SMV increases the fraction of single nucleotide loci that contribute to the total SMV is reduced. This is surprising because the rate of single nucleotide SMV is continues to increase up to the inflection point shown in Fig. 4C and 6C. Although surprising, this may be the result of the overall mutability of single nucleotide repeats. Due to their high polymorphism rate in non-stress conditions, which can be greater than 10^3^ per nucleotide as compared to 10^4^ or greater di and tri – nucleotide MSTs [13, 38-40], any systemic increase in overall MST mutability, such as impaired MMR, will have a greater effect on more stable motifs [38, 39], while having a more blunted effect on single nucleotide MSTs. This was shown, in part, by the Eckert group when they found that impaired MMR causes a similar mutation rate in single, di and for some tetra- nucleotide motifs between 10^3^ and 10^2^ per nucleotide [13, 38-42]. This is underscored by the data when correlating SMV in single nucleotide runs and other MST motifs. Fig. 8C shows a positive slope when plotting single nucleotide run SMV with di and tri-nucleotide SMV. However, the rate increase in single nucleotide repeats is less than for other motifs, based on the slope, which is less than 1 for both motif lengths.

In conclusion, here we found that an expanded definition of MSI, one that includes SMV, may have relevance that can extend beyond CRC, as illustrated here for liver cancer, a cancer type with no known genetic treatment markers. The various implications of SMV on these and other cancers that have not been explored in this paper require further studies.

## METHODS

### TCGA samples

Tumor and extemporaneous tissue control sequences for CRC and LIHC patients were obtained from The Cancer Genome Atlas (http://cancergenome.nih.gov). Patients sequencing data were limited to exomic sequences, which was optimized for exome capture and paired-end 2 × 70+ Illumina sequencing. Further, due to the susceptibility of MST mutation, samples that have undergone genome amplification were not used. A complete list of samples analyzed can be found in supplementary materials (Suppl. Spreadsheet 1).

### Sequencing analysis pipeline

The pipeline was described in more detail in Vaksman et al [18]. However, briefly, TCGA downloaded bam files were reverted to original fastq files using Picard, which were then aligned to HG19/GRCh37 (http://www.genome.ucsc.edu) using BWA-mem [43]. Output sam files were sorted, indexed and filtered for PCR duplicates using samtools, then locally realigned GATK.

### Microsatellite multi-allele software

The MST multi-allele caller is described in greater detail in Vaksman et al [18] however a brief description follows.

A list of MST loci was generated by Tandem Repeat Finder (TRF) [44] using the human reference genome HG19 available on the UCSC genome browser website (http://genome.ucsc.edu). MST genotype and somatic variability for each colorectal and liver cancer patient sequencing data set were evaluated using our multi-allele caller using this MST loci list. Bam files for each patient were used to obtain reads with MSTs through an intermediate step using the Samtools- view command. Reads that did not meet various quality control criteria, such as mapping score below 10% or average phred score below 28 per base, were eliminated by program filters. Determination of MST sequences and sequence lengths was done by alignment of the read to the locus by a user defined minimal length flanking sequence; for this study the defined flanker length was 7 nucleotides on either side of the MST. A MST locus was called based on a user-defined parameter of minimal coverage and an allele is called based on a minimal number of confirming reads. For this study minimal coverage was 15 reads per locus called and a minimum of 3 confirming reads per allele called. Also, the upper limit for coverage was set at 300 reads to remove loci in duplicated regions. Genotype and haplotype for each locus were called based on the following criteria; 1) Loci with a single allele with a minimum coverage of 15 reads were considered homozygotic with no minor alleles. 2) For loci with the appropriate coverage and a second allele, if the allele is 25% of the total depth for the locus or greater than 50% of the depth for the most common allele, this locus would be considered heterozygotic. 3) If an allele does not meet the criteria described in rule 2 or is not the first or second most common allele, yet has at least 3 reads to substantiate this additional allele, it is considered a minor allele or a somatic variation allele. In this paper, SMV rate is defined as the fraction of MST loci with minor alleles [18].

In this project we also analyzed somatic variation in over 3 million non-MST loci and compared the results to SMV. Due to MSTs multi-nucleotide configuration we did not use a nucleotide by nucleotide approach as is commonly used for genotyping, instead we generated over 3 million randomly selected loci consisting of 15-nucleotide long sequences (the approximate mean length of MSTs identified in exome sequencing in our analysis). All the loci used were at least 50 nucleotides away from MST loci. The non-MST loci were analyzed for somatic variability using the multi-allele caller described above with the same user-defined parameters as was done with MST loci [18].

### Statistical analysis

All correlations and regression analyses were done using R and Excel. Plotting presented here was done using Excel table functions for ease of use.
